# Macrosomia predictors and pregnancy outcomes in Gestational Diabetes patients: An observational study from Ha’il, Saudi Arabia

**DOI:** 10.12669/pjms.38.5.5809

**Published:** 2022

**Authors:** Nuzhat Parveen, Naveed Iqbal, Asma Batool, Tariq Mahmoud, Syeda Ali

**Affiliations:** 1 Nuzhat Parveen, Assistant Professor, Department of Obstetrics and Gynecology, College of Medicine, University of Ha’il, Ha’il-81451, Kingdom of Saudi Arabia KSA; 2 Naveed Iqbal, Associate Professor, Department of Obstetrics and Gynecology, College of Medicine, University of Ha’il, Ha’il-81451, Kingdom of Saudi Arabia KSA; 3 Asma Batool, Consultant of Obstetrics and Gynecology, Maternity and children hospital Ha’il, Kingdom of Saudi Arabia; 4 Tariq Mahmoud: Assistant Professor, Department of Obstetrics and Gynecology, College of Medicine, University of Ha’il, Ha’il-81451, Kingdom of Saudi Arabia KSA; 5 Syeda Ali: Associate Professor. Department of Obstetrics and Gynecology, Nishtar Medical University, Multan, Pakistan

**Keywords:** Fetal macrosomia, Gestational diabetes mellitus, HbA1c, Mode of delivery

## Abstract

**Objectives:**

To determine the prevalence, risk factors for macrosomia and pregnancy outcome in women with gestational diabetes (GDM).

**Methods:**

In this prospective observational study, we included the data of 161 pregnant females diagnosed with GDM. The study was conducted from December 1st, 2020 to June 30, 2021, at the Maternity and Children Hospital (MCH) of Hail, Saudi Arabia. The data regarding risk factors of macrosomia was obtained from each patient. The patients were followed till the delivery of the baby. The data regarding the prevalence of fetal macrosomia and its associated outcomes was noted.

**Results:**

The prevalence of fetal macrosomia was 19.8%. Maternal obesity (OR 4.87), poorly controlled diabetes (OR 3.3), previous history of good-sized baby (OR 2.30), previous history of congenital abnormalities (OR 7.2) were the significant risk factors of fetal macrosomia. The prevalence of maternal and fetal complications was high among pregnancies complicated by fetal macrosomia. The prevalence of fetal macrosomia and other fetal complications was high in poorly controlled GDM patients in comparison to optimal control GDM patients.

**Conclusion:**

Fetal macrosomia is a common complication among GDM patients. Maternal obesity and poorly controlled diabetes are the common modifiable maternal factors contributing to macrosomia.

## INTRODUCTION

Gestational diabetes mellitus (GDM) is a worldwide health problem, affecting about 5.0% of all pregnant women.^1^ The reported prevalence varies from country to country or according to the different criteria used for diagnosis of GDM ranging from 1 to >30 percent.^2^ GDM is associated with adverse risks for both the mother and the baby such as higher chances of cesarean delivery, neonatal hypoglycemia, shoulder dystonia, and macrosomia.^3^ Moreover, GDM women are also at higher risk of developing Type-2 diabetes at an early age in life. Hyperglycemia during pregnancy causes fetal adipose tissues disproportionality causing higher body fat, thickening of extremity skin-folds, and an increase in shoulder to head ratio. Due to this changing anthropometry, these babies have a higher risk of shoulder dystocia and fractures.^4^

Fetal macrosomia is defined as if the neonatal weight exceeds >4 Kg, it affects about 10% of the total pregnancies. Macrosomia puts mothers at risk of several complications such as emergency C-section, postpartum hemorrhage, and perianal trauma for mothers, while for babies it increases the chances of fractures of clavicle or humerus bones, birth asphyxia, and shoulder dystocia.^5^,^6^ In comparison to normal weight babies these macrosomia infants have a higher prevalence of morbidities including respiratory distress, meconium aspiration, and mechanical ventilation. Long term complications of macrosomia include a higher prevalence of obesity and diabetes.^7^,^8^

In this present study, we determined the prevalence, risk factors for macrosomia and pregnancy outcome in women with gestational diabetes (GDM).

## METHODS

We performed this prospective observational study, starting from December 1st, 2020 to June 30, 2021, at the Maternity and Children`s Hospital (MCH) of Hail, Kingdome of Saudi Arabia. The largest (250 bedded) tertiary care maternity care setup that provides services to the women and children of Hail province.. Inclusion criteria were pregnant women of Saudi origin, who booked in this facility, identified as GDM during the antenatal period, and delivered at this hospital were included in the study. Exclusion criteria were pregnant women with pre-gestational diabetes (Type- I and II DM) or others with medical (hypertensive) and obstetrical conditions, women of non-Saudis origin, and cases with missing information were excluded. We started this study after getting ethical approval from the Research Ethics Committee of the University of Ha’il [Nr.20455/5/42].

The study questionnaire was sent to the obstetrician working in the hospital for data collection as a google form. Women fulfilling inclusion criteria who delivered during the study period were informed about the purpose of the study and informed consent was taken to include their information. Their information on risk factors and antenatal course of pregnancy was taken from the patient`s health record which was accessed after approval from the hospital. Their delivery details and outcome are recorded at the time of giving birth. All information was entered in forms by the attending physician (a research team member). We included information about the gestational week at which GDM was diagnosed (onset), HbA1c levels during pregnancy, prenatal risks for GDM, antenatal complication, delivery onset, mode of delivery, intrapartum complications, fetal birth weight, birth trauma, APGAR score at birth, and need of Neonatal Intensive Care Unit (NICU) admission.

### Diagnostic criteria for GDM and Cutoff value for HbA1c:

Participating women were screened and diagnosed as GDM according to the NICE recommendations.^9^ A standard oral glucose tolerance test (OGTT), using a 75-gram glucose load was used according to the hospital policy. Fasting and 2-hours Plasma glucose levels were measured to confirm the diagnosis.^10^

For HbA1c, we used values recommended by National Institute for Health and Care Excellence (NICE) guidelines and cut off of 6.1 used. HBA1c value 6.1 or less was considered as normal (well-controlled GDM), while above 6.1 was considered as high (uncontrolled GDM) during pregnancy (taken as a categorical variable).^11^

### Definition of Macrosomia:

Babies weighing 4-kg and above were considered macrosomia. Study participants were distributed into two groups. First included women who delivered a baby weighing 4kg or above, considered as macrosomia. The second group consisted of the deliveries where fetal birth weight was less than 4 kg, non-macrosomia.

### Prenatal Risk Factors:

The pre-pregnancy BMI was calculated for the women by measuring their height in centimeters and pre-pregnancy weight in kilograms. The BMI was calculated by using the formula, Weight in Kg/Height in (m)2 and was analyzed as a categorical variable (Non-Obese: BMI <30 kg/m2, Obese: BMI ≥ 30 kg/m2).^12^

Other prenatal risk factors included in the study are the history of GDM in previous pregnancies, diabetes in first-degree relatives, previous history of intrauterine fetal demise, stillbirths, delivery of congenitally anomalous fetuses, and delivery of good size baby/babies (weighing 4kg or more) before. All responses were recorded in the category of ‘Yes’ or ‘No’.

### Antenatal and Intrapartum Complications:

The information on antenatal complications in the mother (recurrent urinary tract infections (UTIs), pregnancy-induced hypertension (PIH), pre-eclampsia, preterm labor, and development of polyhydramnios) and complications in the fetus (growth restriction, reduced fetal movements, and intrauterine fetal demise) were included. Delivery onset was considered natural if labor started spontaneously and induced where the pregnancy was terminated medically (induction of labor) or surgically (cesarean section) because of pregnancy complications. Mode of delivery included spontaneous vaginal delivery (SVD) and cesarean section (C-section).

Intrapartum complications reported include maternal (shoulder dystocia, extended/3rd-degree perineal tears, and immediate postpartum hemorrhage) and fetal (birth trauma, low APGAR at birth, and need of NICU (Neonatal Intensive Care Unit) admission). Fetal Macrosomia was defined as newborns with birth weight of 4 kilograms or more. Responses for all the variables were recorded in the category of ‘Yes’ or ‘No’.

We used Statistical Package for Social Sciences (SPSS version 23; SPSS Inc., Chicago, IL) for data analysis. Independent-Sample T-test was used to compare means and standard deviations for general characteristics of the study population e.g. age, parity, gestational age at diagnosis, fasting & 2-hours postprandial blood glucose level, HbA1c, pre-pregnancy BMI, and weight gain during pregnancy. Descriptive analysis was done to find the frequency and percentage values for early and late-onset GDM and macrosomia. The relationship of the value of HbA1c to the development of macrosomia was calculated by using cross-tabulation in descriptive statistics. The relationship of time of onset of GDM and fetal macrosomia with prenatal risk factors, antenatal and intrapartum complications was determined through bivariate analysis. P-value <0.05 was taken statistically significant.

## RESULTS

A total of 161 women were diagnosed with GDM during the study period, out of which fetal macrosomia was diagnosed in 32 (19.8%) neonates. Regarding baseline characteristics, the mean parity for Macrosomia was (3.69±2.57) seen while those who delivered babies less than 4kg mean parity was (2.75±2.06) (p=0.036). 2 hours PP BGL was higher in the macrosomia group; 11.01±1.1 versus 9.8±1.9 in normal-weight group, this difference was significant for delivery of babies more than 4 kg (p=0.001) (Table-I).

**Table I:** Data of Baseline Characteristics.

Variables	Fetal Macrosomia (N=32)	Normal weight (N=129)	P-value*
Age of the participants	35.72±5.4	35.30±5.4	0.571
Parity	3.69± 2.57	2.75± 2.06	0.036
Gestational age (weeks) at diagnosis	25.03±8.9	26.03± 7.61	0.240
Fasting BGL	6.6± 0.75	6.3±1.34	0.211
2 hours PP	11.01±1.1	9.8±1.9	0.001
HBA1c	6.7±1.98	6.3± 1.71	0.569
Weight gain during pregnancy	15.1±6.36	13.01±10.1	0.770

History of GDM in previous pregnancies showed a non-significant association for the development of macrosomia. At the same time the women who provide a history of delivering good size babies before, only 19 (27.53%) had macrosomia in this pregnancy while in 50 (72.46%) neonatal weight was found to be less than 4 kg (p-value 0.035). History of late IUFD was not found significant for macrosomia (p-value 0.45). The history of DM in first-degree relatives was also not a significant risk factor (p-value 0.87) (Table-II). The birth weight of the fetus had a significant association with spontaneous labor onset and mode of delivery (Table-III).

**Table II:** Significance of Risk factors in relation to the neonatal birth weight.

Risk factors by history	Fetal Macrosomia (N=32)	Normal weight (N=129)	Odds Ratio (95% CI)	P-value
Obesity	29 (90.6%)	78 (60.5%)	4.87 (1.55-15.29)	0.001
Poorly controlled Diabetes	25 (78.1%)	67 (51.9%)	3.3 (1.33-8.18)	0.007
Advanced Maternal Age (>35 Years)	20 (62.5%)	72 (55.8%)	0.80 (0.42-1.52)	0.49
Previous GDM	16 (50%)	69 (53.5%)	0.87 (0.40-1.88)	0.723
Previous History of Good size babies ≥4kg	19 (59.4%)	50 (38.8%)	2.30 (1.04-5.08)	0.035
Previous history of Congenital abnormalities	06 (18.8%)	04 (3.1%)	7.2 (1.9-27.37)	0.001
Still births	06 (18.8%)	16 (12.4%)	1.63 (0.58-4.56)	0.34
Late IUFD	07 (21.9%)	21 (16.3%)	1.44 (0.55-3.76)	0.45
History of DM in first degree relatives	29 (90.6%)	118 (91.5%)	0.90 (0.23-3.44)	0.87

**Table III:** Association of Fetal macrosomia with onset of labor.

Delivery	Fetal Macrosomia (N=32)	Normal weight (N=129)	P value
**Labor onset**			
Natural onset	12 (37.5%)	27 (21%)	0.045
Medical termination	20 (62.5%)	102 (79%)	
**Mode of delivery**			
SVD	18 (56.2%)	26 (20.2%)	<0.001
Emergency CS	04 (12.5%)	42 (32.6%)	
Elective CS	10 (31.3%)	61 (47.3%)	

CS: Cesarean section.

It’s obvious from the analysis that shoulder dystocia, extended/3rd-degree tear, immediate PPH, and low APGAR score at five minutes had a significant association with birth weight. Odds ratio analysis showed that a low APGAR score at five minutes was significantly associated with macrosomia (p-value 0.002). However, macrosomia (birthweight ≥ 4kg) was non-significant for birth trauma to the fetus (Table-IV).

**Table IV:** Effect of neonatal weight with the Feto-maternal complications.

Feto-maternal complications	Total sample	Normal weight (N=129)	Fetal Macrosomia (N=32)	P-value
Shoulder dystocia	16 (10%)	05 (3.9%)	11 (34.4%)	<0.0001
Extended/3^rd^ degree tear	4(2.5%)	0.0 (0%)	4 (12.5%)	0.001
Immediate PPH	16 (10%)	9 (7.0%)	07 (21.9%)	0.02
Reduced Fetal movements	57 (35.4%)	39 (30.2%)	18 (56.3%)	0.006
Growth restriction	12 (7.5%)	12 (9.3%)	0 (0.0%)	0.07
Birth trauma	3 (2%)	01 (0.8%)	2 (6.3%)	0.04
Low APGAR	13 (8.1%)	03 (2.3%)	10 (31.3%)	<0.001
NICU Admission	20 (12.4%)	10 (7.8%)	10 (31.3%)	<0.001
IUFD	13 (8.1%)	03 (2.3%)	10 (31.3%)	<0.001

IUFD: Intrauterine fetal demise, NICU: Neonatal intensive care unit, PPH: postpartum hemorrhage.

On comparison of the level of control of GDM, analysis shows that intrauterine reduced fetal movements and uterine fetal demise are significantly associated with HbA1c >6.1 during pregnancy (p-value 0.032 and 0.037 respectively). Similarly, High HbA1c is significantly associated with increased prevalence of fetal macrosomia (p-value 0.007). However, it was non-significant for intrauterine growth restriction of the fetus (p-value 0.60). By this analysis, it’s clear that the HbA1c level has high specificity for intrauterine fetal demise and neonatal birth weight (Table-V).

**Table V:** Relationship of Fetal parameters with HbA1c level.

Variables	HbA1c >6.1	HbA1c ≤ 6.1	P-value
Neonatal Birth weight ≥4kg	25 (27.2%)	07 (10%)	0.007
Intrauterine fetal demise (IUFD)	11 (12%)	02 (2.9%)	0.037
Reduced Fetal movements	39 (42.4%)	18 (26.1%)	0.032
Growth restriction	6 (6.5%)	06 (8.7%)	0.60

## DISCUSSION

GDM prevalence is on the rise probably due to the increasing prevalence of elderly pregnant females, obesity, and improvement in antenatal care and GDM detection.^13^,^14^ GDM not only had detrimental effects on mothers’ health, but it also affects the neonates. Blood glucose passes through the placental circulation to the fetus and increases the fetal blood glucose levels resulting in high fetal blood glucose levels.^15^ One of the major comorbidity associated with it is fetal macrosomia.^16^ In this study the prevalence of macrosomia was 19.8%. A recent study by Jenner et al. conducted in Texas among 967 GDM mothers reported macrosomia prevalence of 11.7%.^17^ While a study by Vally et al. from Australia among 202 women with diet-controlled GDM reported macrosomia rate of 7.9% and 5.0% using the two different criteria of macrosomia diagnosis e.g. >90% percentile and >95% percentile of normal weight at 40th week.^18^ A study from Turkey reported a macrosomia rate of 8.6% among non-diabetic mothers.^19^ This prevalence of macrosomia is on the rise, The macrosomia prevalence in the developed world has increased from 5-20% to 15-25% in the last two decades.^20^ Macrosomia prevalence is highly variable, a study including data of 23 different countries reported macrosomia prevalence to vary from 0.5% to 14.9%. Among these developing nations the prevalence was least in India (0.5%).^21^

The risk factors of macrosomia in GDM women in this study were maternal obesity, poorly controlled diabetes, previous history of macrosomia, and history of congenital abnormalities in the previous baby. We did not find any association of advanced age with macrosomia. A study by Said et al. reported maternal weight >80 Kg, maternal age ≥30 years, previous history of fetal macrosomia, and GDM as significant risk factors of macrosomia.^22^ Another recent study has also reported advanced age as the strong predictor of fetal macrosomia.^23^ The difference in this and reported studies is that these studies were conducted on non-diabetic patients while in the present study we only included diabetic patients.

The other fetal complications that occurred in our patients were shoulder dystocia in 10% neonates, low APGAR score in 8.1%, and NICU admission in 12.4% neonates. On comparison of neonatal complications between the macrosomia and non-macrosomia group, the incidence of LOW APGAR score, shoulder dystocia, and reduced fetal movements was significantly high in macrosomia neonates. Regarding maternal complications, the incidence of 3rd-degree tear was higher in the macrosomia group.

We also performed the analysis of fetal complications among women with good control and poor GDM control. The incidence of fetal macrosomia was 27.2% in poorly controlled GDM patients and only 10% in good control GDM. The rate of IUFD and reduced fetal movements was also higher in poorly controlled GDM patients.

### Limitations of the study:

This study has certain limitations, the major limitation is that the number of macrosomia infants was limited, so studies with larger sample sizes are needed to determine the exact prevalence, risk factors, and outcomes of fetal macrosomia among diabetic mothers.

## CONCLUSION

Fetal macrosomia is a common complication among GDM patients. Maternal obesity and poorly controlled diabetes are the common modifiable maternal factors contributing to macrosomia. The outcome of macrosomia is poor in poorly controlled GDM patients in comparison to optimal GDM control. So controlling maternal weight and timely management of GDM can help to reduce the prevalence of fetal macrosomia among GDM mothers.

### Authors’ Contribution:

**NP & NI:** Data collection, study design, manuscript writing, and is responsible and accountable for the accuracy or integrity of the work.

**AB:** Data collection, study design, manuscript drafting.

**TM:** Data collection, analysis and manuscript revision.

**SA:** Did review and final manuscript approval.
